# Retention of Urine

**Published:** 1887-08

**Authors:** 


### RETENTION OF URINE.

DR. John Ashhurst, Jr., Professor of Clinical Surgery in the University of Pennsylvania, in a clinical, lecture, says:

I shall take this opportunity of making a few remarks on the subject of retention of urine. Retention must, in the first place, be distinguished from suppression. The latter is a much graver condition, and depends upon some fault in the kidney, either from organic disease or from sympathy with some other portion of the body. As a result the urine is not secreted, and the waste matters remaining in the blood soon give rise to symptoms of uræmia. In retention the urine is secreted but is not passed, and accumulates in the bladder. If there be free secreetd of urine, and only retention, the patient may live almost indefinitely, particularly when the retention is of a chronic character, such as we meet with in the case of old persons with enlargement of the prostate. In these cases, retention more or less complete may last for many months. Some years ago a patient presented himself at this hospital, with the statement that his urine was running away all the time. He had been under treatment for paralysis of the bladder. On examination, the bladder was found to be greatly distended, the vesical tumor reaching to the umbilicus. There was a certain amount of dribbling, but the bladder had remained full of urine for at least a year. The whole trouble was due to enlargement of the prostate.

Retention of urine may be due to several conditions. It may be the result of spasm. If a person is obliged to hold his water for an unusual length of time, it not unfrequently happens that when he attempts to empty the bladder he finds that he is unable to do so on account of spasm at the neck of the organ. Retention may also depend upon certain general conditions of the system: thus, in typhoid fever retention of urine is a common occurrence. There it is due to the fact that the nervous system, being under the influence of the poison of the disease, fails to recognize the necessity of emptying the bladder. The bladder, under these circumstances, may become much distended and great harm result.

Among the conditions that more commonly come under the observation of the surgeon is what has been termed spasmodic stricture, which is a condition very similar to the spasm at the neck of the bladder already referred to. A person becomes chilled from exposure to cold, and when he attempts to pass water finds that he is unable to do so on account of spasm. There is another condition which is termed congestive retention. Under such circumstances there is probably always some organic change, some stricture, which under ordinary circumstances gives no annoyance; but, as a result of some cause, congestion takes place in the tissues around the stricture, and retention of urine ensues. This appears to have been the condition in the present case. The patient has had a stricture for many years; then, as a result of exposure, there was increased congestion, he was unable to pass his water, and it was found impossible to pass an instrument.

The most important causes of retention of urine are, however, true or organic stricture of the urethra and enlargement of the prostate. It is necessary to distinguish between these, as their treatment differs. Retention from stricture is a more dangerous condition than retention from enlarged prostate. If the patient be a young man, it is probable that the difficulty is not due to enlargement of the prostate, though there may be an acute prostatitis following gonorrhœa which may be accompained by retention. The ordinary form of prostatic hypertrophy is to be looked for in those past middle life. It rarely occurs before the age of fifty years, while stricture is more common between the ages of twenty and fifty years. The age of the patient may therefore enable the surgeon to form an idea as to the cause of the retention. If the retention be not complete or constant, some information may be obtained by observing how the patient passes water. In stricture, although the urine may be passed in a narrow stream, it is passed with a certain amount of force. The propulsive power of the bladder is not impaired, and the urine may be projected through the stricture even more powerfully than under normal circumstances, in accordance with the law of hydraulics that the force of a stream is increased in proportion to the diminution of the caliber of the tube through which it flows. If there be enlargement of the prostate, the urine will dribble from the end of the urethra instead of being passed in the ordinary parabolic curve. In this condition, the cause of the obstruction involves the propulsive power of the bladder.

The use of an instrument is, of course, the most satisfactory method of determining the cause of the retention. If the instrument be arrested in front of the prostatic portion of the urethra, the obstruction is due to stricture, but if it be stopped in the prostatic portion we know that there is enlargement of the the prostate gland. The distance at which the prostate is reached varies in different cases, but the average is six inches. If the obstruction be found at less than six inches from the meatus it is probably due to stricture, but if the distance be more than six inches the prostate gland is probably at fault. As I have already said, it is important to make the distincton between these two conditions, for not only does the treatment vary, but the urgency is also different. If the retention be due to enlargement of the prostate, you may temporize to a certain extent. There is not likely to be such acute distention as will cause rupture of the bladder, and the patient may with safety be allowed to go a certain length of time without active treatment. In stricture, however, the patient is in more imminent danger, and, unless relieved, rupture of the urethra may occur. Rupture of the bladder does not often result from retention due to stricture. If the bladder be distended, and the patient receive a blow over the pubes, rupture may occur, and the urine may escape into the peritoneum, exciting peritonitis, or it may be extravasated into the pelvic cellular tissue. If there be simply distention without violence, it is usually the urethra that gives way. The patient, after a violent straining effort, suddedly feels a sense of relief, the urine passes into the cellular tissue, and after a time gives rise to sloughing and gangrene, requiring free incisions to evacuate the effused fluid.

In retention from enlarged prostate it is usually possible to introduce an instrument without much difficulty, if a proper instrument be employed. The physician is apt to use the catheter found in the pocket-case, which, while sufficiently long for ordinary use, is not long enough to reach the bladder in many cases of prostatic obstruction. I have been called some distance into the country to empty the bladder in a case of retention from enlarged prostate, where the whole difficulty was due to the fact that the instrument employed had been too short. Sometimes there is enlargement of the prostate without obstruction. This occurs when the third lobe is not involved. In almost all instances, if the catheter be sufficiently long and of the proper curve, the retention can be relieved. A good plan is to have one of these long English catheters kept constantly over-curved. The catheter must not only be long, but it must be more than ordinarily curved. In order always to have one in readiness, it is well to keep the stilette over-curved. When the wire is withdrawn, the instrument will retain sufficient curve to pass the obstruction. Another good plan is to mould the catheter to the desired curve by placing it in hot water, and then fixing it in this position by immersing it in cold water. It will then keep the curve sufficiently long to permit the obstruction to be passed. There are special catheters made for these cases of enlarged prostate. Some are made with a large curve, while others have a short angular beak, as in the Mercier's or elbowed catheter. This instrument is straight for nearly its whole lengths, and then has an acute bend. This is sometimes useful, as the sudden rise of the beak enables it to slip over the obstruction.

If you are unable to introduce an instrument in a case of enlarged prostate, it becomes necessary to resort to some operative procedure. Tunneling the prostate has been recommended. This consists in taking a solid instrument, directing it in what is considered the proper direction, and then forcing it through the prostate gland. This is a dangerous procedure, and apt to be followed by hæmorrhage. If you have to resort to any operation, the best plain is to tap the bladder. If the prostate be not large this can be done through the rectum, but if the prostate be much increased in size it is safer to tap above the pubis. This may be done very conveniently with the aspirator, but this has the disadvantage that the operation will probably have to be repeated as often as the bladder refills. It sometimes happen, however, that within a few hours after a patient has been relieved he is able to pass his water in the natural way. This also occurs after the use of the catheter, and the patient may go a considerable time without again requiring its aid.

Where the obstruction is due to stricture, and it is found impossible to pass an instrument, the urethra may be opened behind the point of obstruction. In this case I tapped the urethra at the apex of the prostate gland. Stricture, as you know, never involves the prostatic portion of the urethra, but is usually seated at the junction of the bulbous and membranous portions. If, therefore, an opening is made in the urethra at the apex of the prostate gland, you are certain to get behind the stricture. Such an operation is of course not adapted to retention due to enlargement of the prostate, although even there it may be employed to facilitate the introduction of a catheter. In performing the operation, an incision about three-quarters of an inch in length is made in the median line of the perineum, beginning half an inch in front of the anus. The forefinger of the left hand is introduced into the rectum and placed at the apex of the prostate, which can be readily recognized. The knife is then inserted into the incision, with its back towards the anus, and, guided by the finger in the rectum, is pushed onward until its point reaches the apex of the prostate gland. Having reached that point, you cut forward. This opens the urethra, which is at once known by the gush of urine. The knife is then withdrawn and a grooved director passed into the bladder, when, if the opening is not found to be sufficient, it may be cautiously enlarged. An elastic catheter is next introduced upon the director and secured.

The operation was performed in this case five days ago. We shall now remove the tube for the first time, and insert a clean catheter. The urine is beginning to escape by the side of the tube, and in a few days we shall dispense with it entirely. In the course of another week the urethra may be in such a condition as to permit the passage of an instrument, although when the operation was performed this was impossible. If a sound can be passed, we shall be able to cure the stricture by simple dilatation. If we are still unable to pass the stricture, I shall submit to the patient whether he desires an operation for the cure of the stricture, or whether he is satisfied to pass his water through the perineum. Many patients prefer the latter alternative, which is attended with no particular inconvenience. The sphincter power of the bladder is of course preserved, so that there is no incontinence. The only inconvenience is that in urinating the individual must assume a sitting posture. I have had patients who have submitted to an operation for the relief of the stricture, but, finding a tendency to recontraction requiring the constant introduction of instruments, have preferred to abandon the urethra and pass water through the perineum.

The operation which I have described is adapted to cases of retention of urine due to stricture, and not to those due to any other condition. When the retention is due to enlarged prostate, and a catheter cannot be passed, the bladder must be opened either behind the prostate or above the pubis. For the simpler forms of retention from spasm, it is usually sufficient to put the patient in a warm bath and give an anodyne. If this is not successful, a remedy which was formerly much employed may be tried. This is the tincture of the chloride of iron. This has the effect of relieving spasm, and has been found to be very successful. Where you can do it, however, it is always better to relieve the retention at once by the use of the catheter, a soft instrument being preferred. It is not desirable to have the retention last even a few hours, for there is always a certain amount of damage done to the ureters and kidneys even in this short period.—*Quarterly Compendium of Medical Science.*

